# Case Report: Sirolimus Alleviates Persistent Cytopenia After CD19 CAR-T-Cell Therapy

**DOI:** 10.3389/fonc.2021.798352

**Published:** 2021-12-23

**Authors:** Limin Xing, Yihao Wang, Hui Liu, Shan Gao, Qing Shao, Lanzhu Yue, Zhaoyun Liu, Huaquan Wang, Zonghong Shao, Rong Fu

**Affiliations:** Department of Hematology, General Hospital, Tianjin Medical University, Tianjin, China

**Keywords:** sirolimus, chimeric antigen receptor T cell, cytopenia, CD19, diffuse large B-cell lymphoma

## Abstract

Chimeric antigen receptor T (CAR-T) cells show good efficacy in the treatment of relapsed and refractory B-cell tumors, such as acute B-cell leukemia (ALL) and diffuse large B-cell lymphoma (DLBCL). The main toxicities of CAR-T include cytokine release syndrome, immune effector cell-associated neurotoxicity syndrome, cytopenia, and severe infection. It is still very difficult for CAR-T to kill tumor cells to the maximum extent and avoid damaging normal organs. Here, we report a case of DLBCL with persistent grade 4 thrombocytopenia and severe platelet transfusion dependence treated with CD19 CAR-T cells. We used sirolimus to inhibit the sustained activation of CAR-T cells and restore normal bone marrow hematopoiesis and peripheral blood cells. Moreover, sirolimus treatment did not affect the short-term efficacy of CAR-T cells, and DLBCL was in complete remission at the end of follow-up. In conclusion, sirolimus can represent a new strategy for the management of CAR-T cell therapy-related toxicity, including but not limited to hematotoxicity. However, further controlled clinical studies are required to confirm these findings.

## Introduction

Chimeric antigen receptor T cell (CAR-T) therapy is a new type of tumor immunotherapy. Gene modification enables T cells to express tumor-associated antigen receptors and intracellular signal transduction domains connected with them, ultimately recognizing and removing tumor cells. Anti-CD19 CAR-T therapies have been shown to have high levels of efficacy in patients with relapsed or refractory diffuse large B-cell lymphoma (1–4).

However, CAR-T therapy has unique and serious toxicities, including cytokine release syndrome (CRS), immune effector cell-associated neurotoxicity syndrome (ICANS), hemophagocytic lymphohistiocytosis (HLH), and cytopenia, which can lead to acute progressive respiratory and circulatory failure, severe brain injury, and even death (5, 6).

Cytopenia usually occurs in the first month after CAR⁃T therapy but can last for more than 3 months in some patients. Persistent cytopenia can lead to severe infection and bleeding, endangering the lives of patients. In addition to supportive therapies, such as red blood cell transfusion and platelet transfusion, the main therapeutic methods include the use of granulocyte colony-stimulating factor (G-CSF) and thrombopoietin (TPO) receptor agonist. However, the above treatments still cannot alleviate CAR-T therapy-induced cytopenia in few patients (7, 8).

Sirolimus has antifungal, antitumor, antiproliferative, and immunosuppressive effects and is an effective inhibitor of antigen-induced proliferation of T and B cells and antibody production (9, 10).

Here, we report a case of sustained hemocytopenia after CAR-T therapy, which was relieved after sirolimus treatment.

## Case Report

### Diffuse Large B-Cell Lymphoma History

The patient was a 55-year-old man. In February 2017, the patient developed fatigue, with no fever or night sweats. In August 2017, the symptoms worsened, and CT examination in the local hospital indicated bilateral adrenal tumor lesions. On September 7, 2017, positron emission tomography-computed tomography (PET-CT) showed multiple soft tissue density masses in bilateral adrenal glands (4.1 cm × 3.0 cm on the left and 7.2 cm × 3.9 cm on the right), abnormally increased metabolism (SUVmax: 18.3 on the left and 17.8 on the right), and splenomegaly, and further diagnosis of PET-CT results revealed lymphoma. On September 12, 2017, a biopsy of the right adrenal tumor was performed, showing CD20 (+), EMA (weak +), Ki-67 70–80%, CD3 (−), CGA (−), and a pathological diagnosis of diffuse large B-cell lymphoma (DLBCL). A bone marrow smear showed lymphoma involving the bone marrow. Therefore, this patient was diagnosed with DLBCL stage IVEA, International Prognostic Index (IPI) score of 3, age-adjusted (aa) IPI score of 3, and considered at high-risk. Thirty years before, he had undergone binocular strabismus surgery. Ten years later, he had a thoracic vertebral compression fracture, and he had a history of hypertension for the past 10 years.

The patient received six cycles of Rituximab, Cyclophosphamide, Adriamycin, Vincristine, and Prednisone chemotherapy. After chemotherapy, the efficacy was evaluated as a partial response (PR).

From June 5, 2018 to July 13, 2018, the patient received bilateral intensity-modulated radiotherapy on bilateral adrenal glands with a prescription dose of 95% PTV 50 Gy/2 Gy/25 F. Then, a Rituximab, Gemcitabine, and Oxaliplatin regimen was administered on January 3, 2020, February 10, 2020, and March 13, 2020.

On December 6, 2020, an abdominal CT showed bilateral adrenal masses, hepatogastric space, and multiple enlarged retroperitoneal lymph nodes, which were considered as indicators of progression of lymphoma according to the patient’s history. On December 21, 2020, a CT-guided puncture biopsy of the retroperitoneal tumor was performed. The pathological findings showed a DLBCL non-GCB subtype. Therefore, this patient was diagnosed with relapse DLBCL stage IVEA, with an IPI score of 5 and aaIPI score of 3, and considered to be at high-risk.

### CAR-T Cell Therapy

Since previous reports showed that CAR-T-CD19 had a good curative effect in the treatment of refractory and recurrent DLBCL, the patient was enrolled in the clinical trial (NCT03994913) with the approval of the ethics committee of the General Hospital of Tianjin Medical University (IRB2019-181-01) and with the patient’s informed consent.

In brief, T lymphocytes were isolated from the peripheral blood of the patient and amplified *in vitro*, after which CAR-T cells were generated by lentiviral transduction. The humanized anti-CD19 auto CAR-T cell injection was composed of humanized CD19 single-chain antibody (scFv) huHD37 and a CD8a hinge region, a CD8a transmembrane region, 4-1BB, and CD3, connected in sequence.

**Figure 1:**
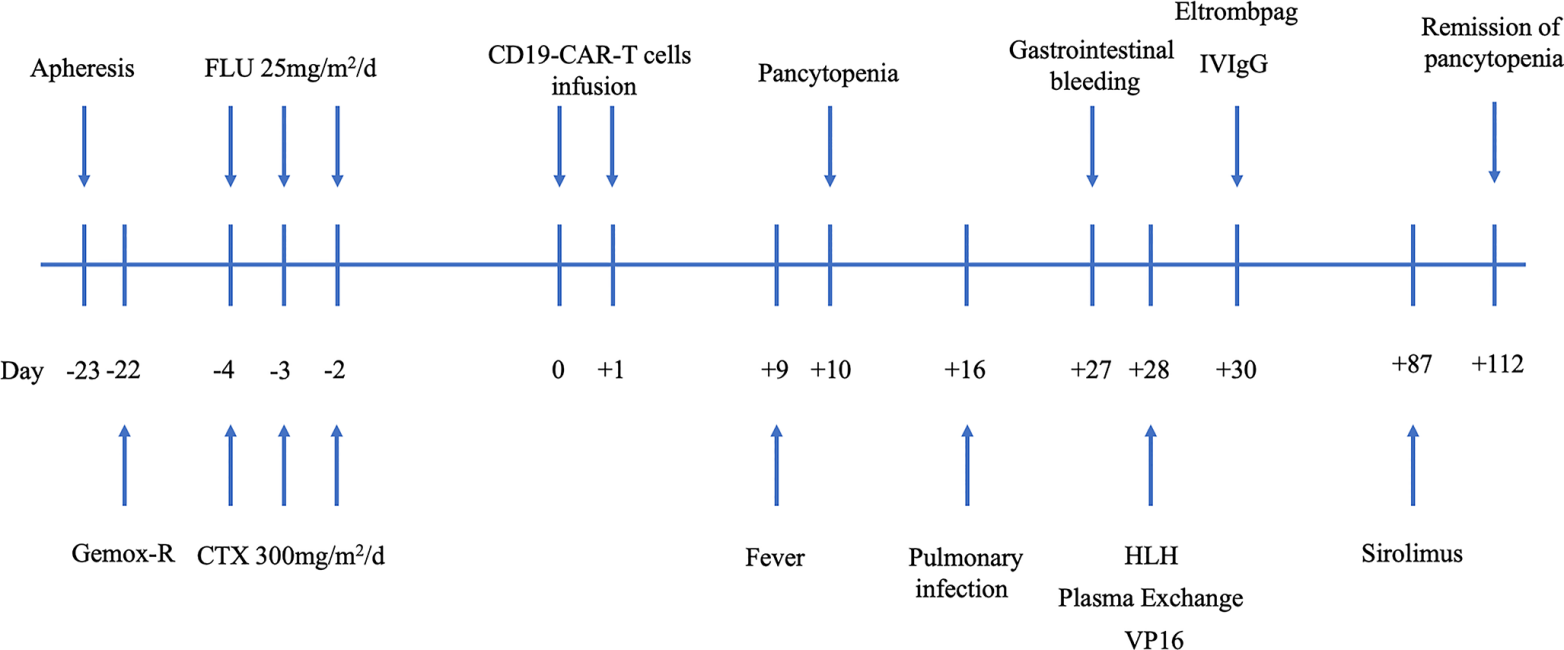
Procedure of CD19 CAR-T cell manufacture and the clinical application scheme. CAR-T, chimeric antigen receptor T cell; CTX, cyclophosphamide; Gemox-R, Gemcitabine, Oxaliplatin, and Rituximab; HLH, hemophagocytic lymphohistiocytosis; VP16, etoposide.

The FC regimen (fludarabine 25 mg/m^2^/day D-4 to D-2 and CTX 300 mg/m^2^/day D-4 to D-2) was used to deplete lymphocytes. In total, 3 × 10^8^ CAR-T cells were infused in the patient for 2 consecutive days (D0 and D1). The infusion process was smooth, and the patient had no obvious adverse reactions (**Figure 1**).

### Related Toxicity Management of CD19 CAR-T Therapy

#### CRS and ICANS

On the ninth day after infusion, the patient developed fever, with the highest temperature being 39.1°C, along with shivering, headache, arthralgia, anorexia, and gastrointestinal discomfort. The patient was diagnosed with CRS (grade 2) (11) and treated with tocilizumab and dexamethasone. The patient’s symptoms were relieved after 4 days.

#### Pulmonary Infection

On the 16th day, the patient developed fever again with cough and suffocation. The patient was diagnosed with a pulmonary infection and treated with meropenem and linezolid. Thereafter, the patient had repeated fever and received a variety of antibiotics, including teicoplanin, tigecycline, cefoperazone/sulbactam, sulfamethoxazole/trimethoprim, voriconazole, and penciclovir.

#### HLH

On the 28th day, the patient had a persistent fever and pancytopenia. Hemophagocytosis was observed in the bone marrow. Fibrinogen level was <1.5 g/L, and ferritin was 8,029.94 mg/L. The expression of CD107 of in natural killer (NK) cells was decreased. The soluble CD25 level was 30,902 U/ml. HLH was diagnosed according to the HLH 2004 criteria (12). The patient was treated with etoposide, dexamethasone, liposome adriamycin, intravenous immunoglobulin (IVIgG), and plasma exchange.

#### Cytopenia

From day 10, thrombocytopenia, anemia, and neutropenia persisted in this patient. In addition to the above treatment, we treated the patient with platelet infusion, red blood cell infusion, recombinant human G-CSF, recombinant human erythropoietin (EPO), recombinant human TPO, and TPO receptor agonists Eltrombopag and Avatrombopag.

### Sirolimus Successfully Alleviates Persistent Pancytopenia

Although the patient had been treated with the abovementioned treatments, there was persistent pancytopenia, especially severe thrombocytopenia, which required platelet transfusion daily. The patient had gastrointestinal bleeding, hematochezia, hematemesis, oral blisters, gingival bleeding, systemic skin, and mucosal bleeding points, purpura and ecchymosis, and hematuria.

**Figure 2:**
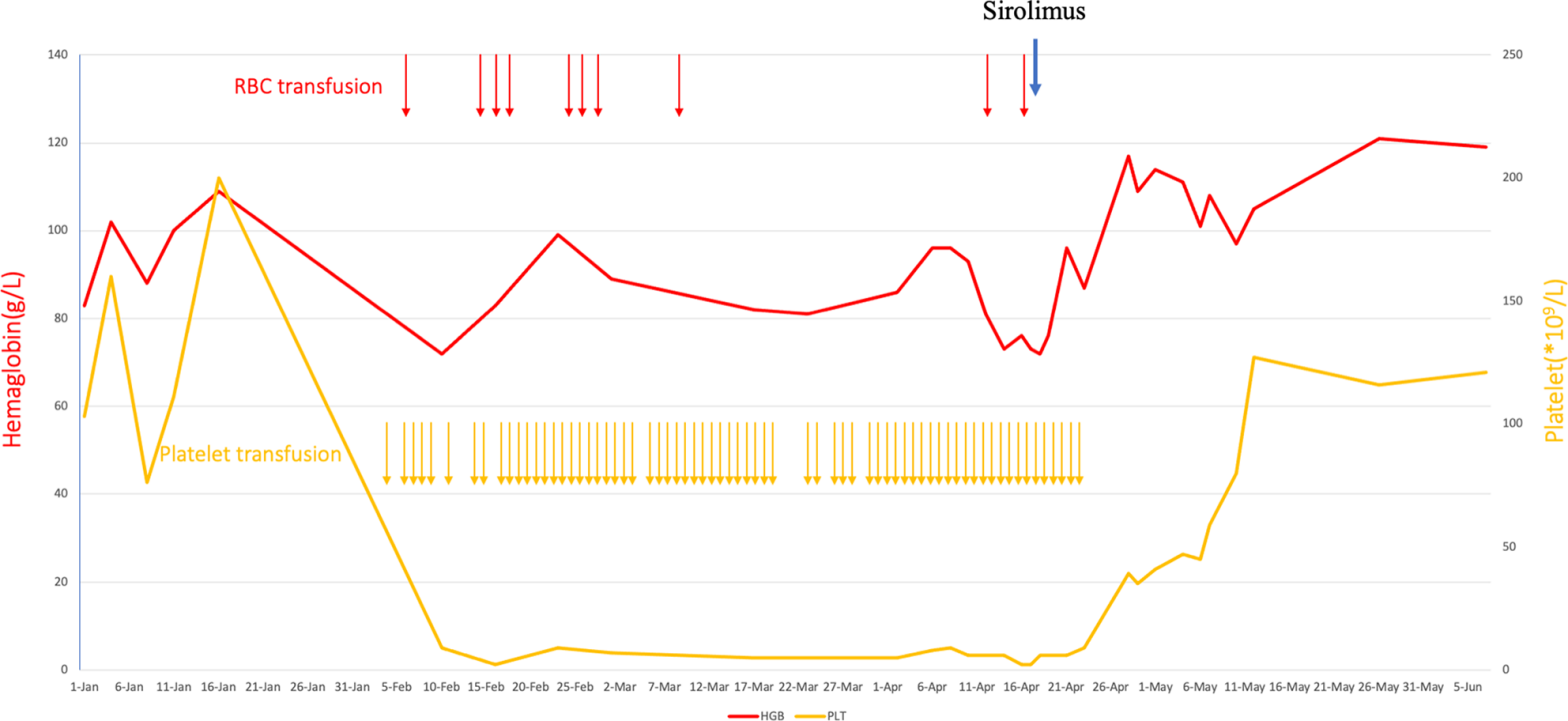
Hematological adverse events and recovery after anti-CD19 CAR-T-cell therapy and sirolimus for r/r DLBCL.

Starting from day 87, the patient was treated with sirolimus (Huabei Pharmaceutical Company, China). The initial dose of sirolimus was 0.5 mg/day. The dose was increased by 0.5 mg every 2–3 days. The maximum dose was 2.5 mg/day. On the sixth day after sirolimus treatment, the patient was no longer dependent on platelet transfusion and red blood cell transfusion. On the 12th day after sirolimus treatment, the platelet level increased to 39 × 10^9^/L, and the level of hemoglobin increased to 117 g/L. On the 25th day after sirolimus treatment, the platelet count increased to normal levels. Subsequently, the platelet count and hemoglobin levels continued to be normal. After the platelet level of the patient returned to normal levels, sirolimus dose was gradually decreased by 0.5 mg every 3 days and finally stopped (**Figure 2**; **Supplementary Figure 1**; **Supplementary Data**).

### Efficacy Evaluation of CD19-CAR-T Therapy

**Figure 3:**
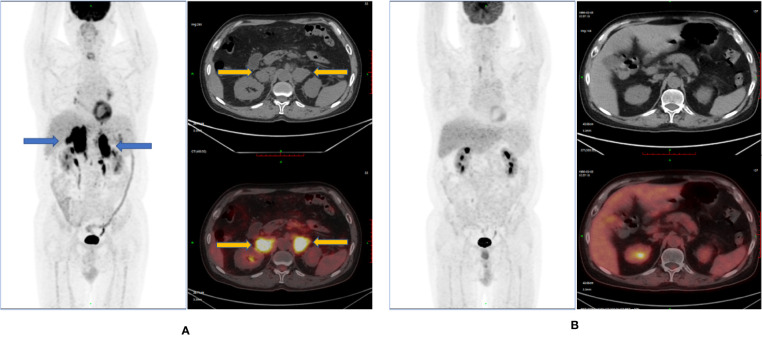
Remission status r/r DLBCL after anti-CD19 CAR-T-cell therapy and sirolimus. **(A)** Full-body ^18^FDG-PET scan pre- anti-CD19 CAR-T-cell therapy. **(B)** full-body ^18^FDG-PET scan post- anti-CD19 CAR-T-cell therapy.

On December 14, 2020, PET-CT showed the following: (1) soft tissue density masses and abnormal metabolism were found in the bilateral adrenal glands; (2) abdominal multiple enlarged lymph node shadows were present, with an abnormal increase in metabolism, considered as tumor invasion; (3) the right lobe of the liver appeared to have a low-density mass shadow, and multiple irregular nodular thickening of the peritoneum was observed, with an abnormal increase in metabolism, considered as tumor invasion; (4) the metabolism of the body and tail of the pancreas was abnormally increased, likely due to be tumor invasion; and (5) based on the above observations and the history of the disease, progression of the disease was considered. The Deauville score was 5 points (**Figure 3A**).

On January 14, 2021 (-6d), PET-CT showed nodule shadows and abnormal increases in metabolism in the bilateral adrenal multiple soft groups, which was consistent with the image changes after lymphoma treatment. Combined with the medical history, effective treatment and partial remission were considered. The Deauville score was 4 points.

On March 13, 2020 (+52d), PET-CT showed no sign of a malignant tumor on body imaging. Combined with the patient’s history, it was consistent with the image changes in lymphoma after treatment. Effective treatment and remission were also considered. The Deauville score was 3 points.

On May 6, 2020 (+106d), PET-CT showed no sign of a malignant tumor on body imaging. The Deauville score was 1 point (**Figure 3B**).

## Discussion

After CAR-T treatment, some patients develop cytopenia, most of which appears in the first month, and remission. A small number of patients can have persistent cytopenia for more than 3 months, and a few patients cannot even fully recover (13). The causes of cytopenia include damage to bone marrow hematopoiesis caused by previous administration of chemotherapeutic drugs, chemotherapeutic drugs for depleting lymphocytes, the abnormal immune activation caused by infusion of CAR-T cells, and the immune damage of bone marrow hematopoietic stem progenitor cells and the hematopoietic microenvironment (1–4, 14, 15).

Symptomatic supportive treatments, such as red blood cell (RBC) transfusion, platelet transfusion, and infection control and prevention, are the fundamental methods of treatment for severe cytopenia (6, 16). In patients with neutropenia, G-CSF can effectively increase the level of neutrophils without affecting the efficacy of CAR-T cells or increasing the toxicity of CAR-T cells (17, 18). In patients with thrombocytopenia, glucocorticoids, IVIgG, rhTPO, and TPO receptor agonists, such as eltrombopag, romiplostim, and avatrombopag, are commonly used (6, 13, 19–21). In patients with long-term persistent two- or three-lineage cytopenia, successful cases of autologous hematopoietic stem cell infusion and allogeneic hematopoietic stem cell transplantation have also been reported (22, 23).

Our patient in this report was treated with dexamethasone, IVIgG, rhTPO, eltrombopag, and avatrombopag. The platelet and hemoglobin levels continued to decrease, especially the platelet level, which continued to be <10 × 10^9^/L, accompanied by active bleeding. CAR-T cells tests showed that persistent proliferation of CAR-T cells. We speculated that the abnormal proliferation of CAR-T cells led to bone marrow failure, resulting in pancytopenia. Therefore, we decided to use drugs to block the proliferation of CAR-T cells.

Antithymocyte globulin and cyclosporine are the most commonly used drugs for the treatment of immune bone marrow failure. However, considering that they may lead to longer-term cytopenia and immunosuppression, increase the chance of severe infection, and promote the deterioration of lymphoma, we selected sirolimus.

Previous studies have confirmed that sirolimus is effective in the treatment of patients with autoimmune hemocytopenia, such as aplastic anemia (24, 25), immune thrombocytopenia (26–29), autoimmune hemolytic anemia (30–32), pure red cell aplastic anemia (33, 34), autoimmune lymphoproliferative syndrome (35, 36), and systemic lupus erythematosus (35, 37). Our experience shows that sirolimus is effective and well tolerated in the treatment of bone marrow failure caused by CAR-T cells.

Our study has some limitations. First, there was only one patient, and the possibility of spontaneous bone marrow recovery could not be ruled out. Second, the observation time was short, and the long-term effect of sirolimus on the CAR-T cell treatment of lymphoma could not be obtained.

In conclusion, our case report shows the effects of sirolimus in a patient with cytopenia after CAR-T cell therapy, particularly in case of severe thrombocytopenia. Further studies, especially controlled randomized trials, are needed and may help determine the effects of the use of sirolimus in such patients. Our study also shows that sirolimus can inhibit the proliferation of CAR-T cells, and further study of its mechanism and role in the regulation of CAR-T will help to accurately control CAR-T cell therapy and avoid the occurrence of fatal CRS, ICANS, and cytopenia.

## Data Availability Statement

The original contributions presented in the study are included in the article/**Supplementary Material**. Further inquiries can be directed to the corresponding authors.

## Ethics Statement

The studies involving human participants were reviewed and approved by the approval of ethics committee of General Hospital of Tianjin Medical University (IRB2019-181-01). The patients/participants provided their written informed consent to participate in this study.

## Author Contributions

HW and LX collected and analyzed data and wrote the manuscript. YW, HL, SG, QS, LY, ZL, ZS, and RF collected and analyzed data. All authors contributed to the article and approved the submitted version.

## Conflict of Interest

The authors declare that the research was conducted in the absence of any commercial or financial relationships that could be construed as a potential conflict of interest.

## Publisher’s Note

All claims expressed in this article are solely those of the authors and do not necessarily represent those of their affiliated organizations, or those of the publisher, the editors and the reviewers. Any product that may be evaluated in this article, or claim that may be made by its manufacturer, is not guaranteed or endorsed by the publisher.

## Supplementary Material

Supplementary Figure 1 The peripheral blood CAR-T copies and the counts of white blood cells after anti-CD19 CAR-T-cell therapy and sirolimus for r/r DLBCL.
