# Supplementary figures and images for: Case Report: Sirolimus Alleviates Persistent Cytopenia After CD19 CAR-T-Cell Therapy
Source: Front Oncol. 2021 Dec 23;11:798352. doi: 10.3389/fonc.2021.798352 (PMC8733571; doi:10.3389/fonc.2021.798352)

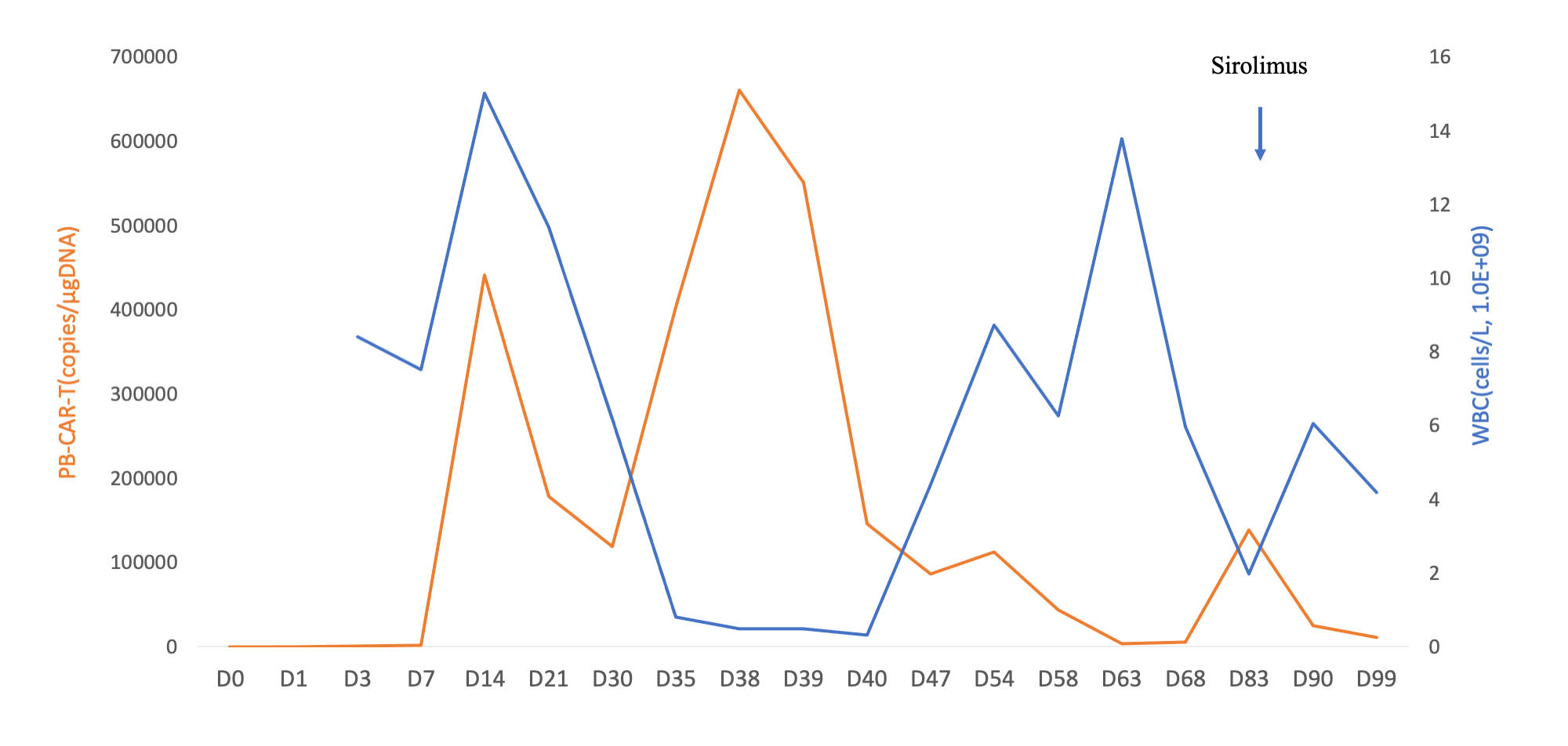

Supplement: Supplementary Figure 1 — The peripheral blood CAR-T copies and the counts of white blood cells after anti-CD19 CAR-T-cell therapy and sirolimus for r/r DLBCL. [file Image_1.tiff]
